# Supplementary material for: Reconciling Mining with the Conservation of Cave Biodiversity: A Quantitative Baseline to Help Establish Conservation Priorities
Source: PLoS One. 2016 Dec 20;11(12):e0168348. doi: 10.1371/journal.pone.0168348 (PMC5173368; doi:10.1371/journal.pone.0168348)
Supplement: S1 Dataset — (ZIP) [file pone.0168348.s002.zip › Taxa/Serra Norte/SN_2013/N5S/Lista N5S-80.pdf]

CAVIDADE N5S-0080

Classe	Ordem	Fam/Outros	Gên/Outros	Espécie	S-U	U-S	Zon.
Arachnida	Amblypygi	Phrynidae	*Heterophrynus*	*longicornis*		4	P
Arachnida	Araneae	Araneidae	*Alpaida*	aff. *negro*	X		P
Arachnida	Araneae	Ctenidae		jovem	2		E/P
Arachnida	Araneae	Pholcidae	*Mesabolivar*	*aurantiacus*	X		P
Arachnida	Araneae	Pisauridae		jovem		2	P
Arachnida	Araneae	Theridiosomatidae	*Plato*	sp.1	X	X	P
Arachnida	Opiliones	Sclerosomatidae	*Prionostema*	sp.1	1	1	P
Arachnida	Opiliones	Stygnidae	*Protimesius*	*gracilis*	2		P
Diplopoda	Polydesmida	Fuhrmannodesmidae		jovem		X	E
Diplopoda	Spirostreptida	Pseudonannolenidae	*Pseudonannolene*	jovem		1	E
Entognatha	Collembola	Paronellidae		sp.1		X	E
Insecta	Blattodea			jovem	1	2	E/P
Insecta	Coleoptera	Carabidae		sp.2		X	E
Insecta	Coleoptera	Carabidae		sp.10	X		E/P
Insecta	Coleoptera	Ptiliidae		sp.1		X	E
Insecta	Coleoptera	Staphylinidae		sp.4	X	X	E
Insecta	Coleoptera	Staphylinidae		sp.5		X	E
Insecta	Diptera	Psychodidae	Phlebotominae	sp.	X		P
Insecta	Diptera	Tipulidae	Tipulinae	sp.		X	
Insecta	Heteroptera	Veliidae	*Paravelia*	sp.		X	P
Insecta	Homoptera	Cixiidae		jovem	X		E
Insecta	Hymenoptera	Formicidae	*Paratrechina*	sp.1		X	E
Insecta	Hymenoptera	Formicidae	*Paratrechina*	sp.2	X		P
Insecta	Hymenoptera	Formicidae	*Trachymyrmex*	sp.1	X		E
Insecta	Lepidoptera	Noctuoidea		sp.5	1		P
Insecta	Mantodea	Mantidae		sp.1	1		P
Insecta	Orthoptera	Phalangopsidae	*Paraclodes*	sp.	3		E
Insecta	Orthoptera	Phalangopsidae	*Phalangopsis*	sp.	2	3	P
Gastropoda	Pulmonata	Systrophiidae	*Happia*	sp.		X	E
Symphyla		Scutigerellidae		sp.		X	E
Amphibia	Anura			sp.	1		P
Mammalia	Chiroptera	Emballonuridae	*Peropteryx*	*kappleri*	1	1	P
Mammalia	Chiroptera	Emballonuridae	*Peropteryx*	*macrotis*		1	P
